# The Influence of Temperature in the Wire Drawing Process on the Wear of Drawing Dies

**DOI:** 10.3390/ma17204949

**Published:** 2024-10-10

**Authors:** Maciej Suliga, Piotr Szota, Monika Gwoździk, Joanna Kulasa, Anna Brudny

**Affiliations:** 1 Faculty of Production Engineering and Materials Technology, Czestochowa University of Technology, Armii Krajowej 19, 42-201 Czestochowa, Poland; piotr.szota@pcz.pl (P.S.); monika.gwozdzik@pcz.pl (M.G.); 2 Łukasiewicz Research Network–Institute of Non-Ferrous Metals, Sowińskiego 5, 44-100 Gliwice, Poland; joanna.kulasa@imn.lukasiewicz.gov.pl (J.K.); anna.brudny@imn.lukasiewicz.gov.pl (A.B.)

**Keywords:** wire drawing, steel, die wear, FEM, tungsten carbide, tribology, friction

## Abstract

This paper presents a wear analysis of tungsten carbide drawing dies in the process of steel wire drawing. The finite element method (FEM) analysis showed a significant correlation between drawing die geometry, single reduction size and drawing speed on the rate of drawing die wear. It has been shown that in steel wire drawing at higher drawing speeds, intense heating of the drawing die occurs due to friction at the wire/drawing die interface, leading to premature wear. Tribological tests on the material for the drawing die cores (94%WC+6%Co) confirmed the gradual abrasion of the steel and carbide sample surfaces with the “products” of abrasion sticking to their surfaces. The increase in temperature increases the coefficient of friction, translating into accelerated wear of the drawing dies.

## 1. Introduction

The drawing die is the basic tool of the drawing process, and it has a decisive influence on the process parameters and the properties of the steel wires [1,2]. Cemented carbides, synthetic diamonds, and cemented carbides with a ceramic coating are used to manufacture drawing dies [3,4,5]. Currently, millions of tons of wire and wire products (ropes, springs, screws, anchors, nails, fasteners, etc.) are produced in the world every year using the drawing method. These products are used in many branches of the economy (including construction, machine industry, and mining).

Drawing technology is the determining factor for using a particular type of drawing dies. The drawing die is built with a steel casing to protect the drawing die core from mechanical damage. In steel wire wet drawing, drawing dies with tungsten carbide or diamond inserts are used, while special ceramic coatings are also applied to the tungsten carbide surfaces to increase their durability. On the other hand, in dry wire drawing, with wire diameters over 1 mm, tungsten carbide inserts are usually used for financial reasons. Drawing dies used in wire manufacturing are also classified according to the shape of the drawing part, i.e., concave, convex sigmoid, curved and conical dies. Drawing dies can also be divided according to the drawing method, i.e., conventional, hydrodynamic [6,7], and roller dies [8,9]. Hence, when designing the drawing process, a specific type of drawing die is selected based on the surface treatment of the wire (mechanical descaling, pickling) [10,11,12], the application of lubricant layers, the type of wire drawing machine (dry drawing, wet drawing), the wire diameter, and the number of draws [13,14,15].

Depending on the type of drawing die, its lifespan varies from a few to more than 100 tonnes of processed material. The most common type of drawing die in the wire industry is the conical die, which consists of a steel casing and a tungsten carbide core. High pressures in the contact area between the material and the drawing die cause the drawing die to wear, cracks on its surface, and changes in geometry. The wear rate of drawing dies depends largely on the drawing technology. The basic parameters of the drawing process are drawing geometry, distribution of single and total reduction, degree of wire strengthening, steel grade, lubrication conditions [16,17], and drawing speed [18]. The drawing speed is one of the main factors that can affect the wear rate of drawing dies. In multi-stage drawing, an increase in drawing speed generates a large amount of heat at the wire/drawing die interface, leading to a significant rise in temperature, which, on the one hand, contributes to a deterioration in lubrication conditions and an increase in friction and, on the other hand, reduces the wear resistance of the carbide. Hence, in industrial practice, drawing die wear is common after drawing 1 tonne of wire.

Today, an experimental approach can be used to analyse the wear of drawing dies by measuring the wear and the change in geometry of the drawing dies after the drawing process depending on the amount of material drawn and the drawing conditions [19,20], tribological studies of the materials used for drawing dies and computer simulations of the drawing process (FEM) [21,22,23,24,25]. The design of metal forming tools is a complex, time-consuming, and resource-intensive issue. Hence, the combined experimental and numerical analysis methods are currently used for optimisation.

This paper presents an analysis of the influence of drawing process parameters, i.e., drawing speed and angle, single reduction size, on wire heating, stress distribution and drawing die wear. On the other hand, tribological studies made it possible to determine the relationship between temperature and the friction coefficient and wear of the steel/carbide friction pair.

## 2. Materials and Methods

### 2.1. Computer Simulations of the Wire Drawing Process

The study involved numerical modelling of the wire drawing process using the Simufact 2023.2 commercial computer program (Hexagon, Hamburg, Germany). The program uses the finite element method to solve the computational problem. The wire drawing process was axisymmetric in a plane deformation condition. A total of 3600 flat tetrahedral elements with an average mesh edge size of 0.15 mm were used for the calculations.

The MARC solver developed based on the displacement method was used for the calculations. The MARC methodology is based on the stiffness of the system and is based on the force–displacement relationship (1):

(1) K⋅u=f

where *K* is the system stiffness matrix, *u* is the nodal displacement and *f* is the force vector.

Once the displacement vector *u* is determined, the deformations in each element can be calculated based on the displacement (2):

(2) $$\varepsilon_{el}=\beta \cdot u_{el}$$

The stress in an element is determined based on the stress–deformation relationship (3):

(3) $$\sigma_{el}=L\cdot \varepsilon_{el}$$

where $\sigma_{el}$ is the stress and $\varepsilon_{el}$ is the deformation in the elements, and $u_{el}$ is the displacement vector associated with the element nodal points; and *β* and *L* are the deformation–displacement and stress–deformation relations, respectively.

The hardening curve taken from the Simulfact 2023.2 program database, equation $\sigma (\varepsilon ,\dot{\varepsilon },T)$ (4), was used to simulate the drawing.

(4) $$\sigma =C_{1}\cdot e^{\left(C_{2}\cdot T\right)}\cdot \varepsilon^{\left(n_{1}\cdot T+n_{2}\right)}\cdot e^{\left(\frac{l_{1}\cdot T+l_{2}}{\varepsilon }\right)}\cdot {\dot{\varepsilon }}^{\left(m_{1}\cdot T+m_{2}\right)}$$

where T—temperature, ε—true strain, and $\dot{\varepsilon }$—strain rate.

**Figure 1:**
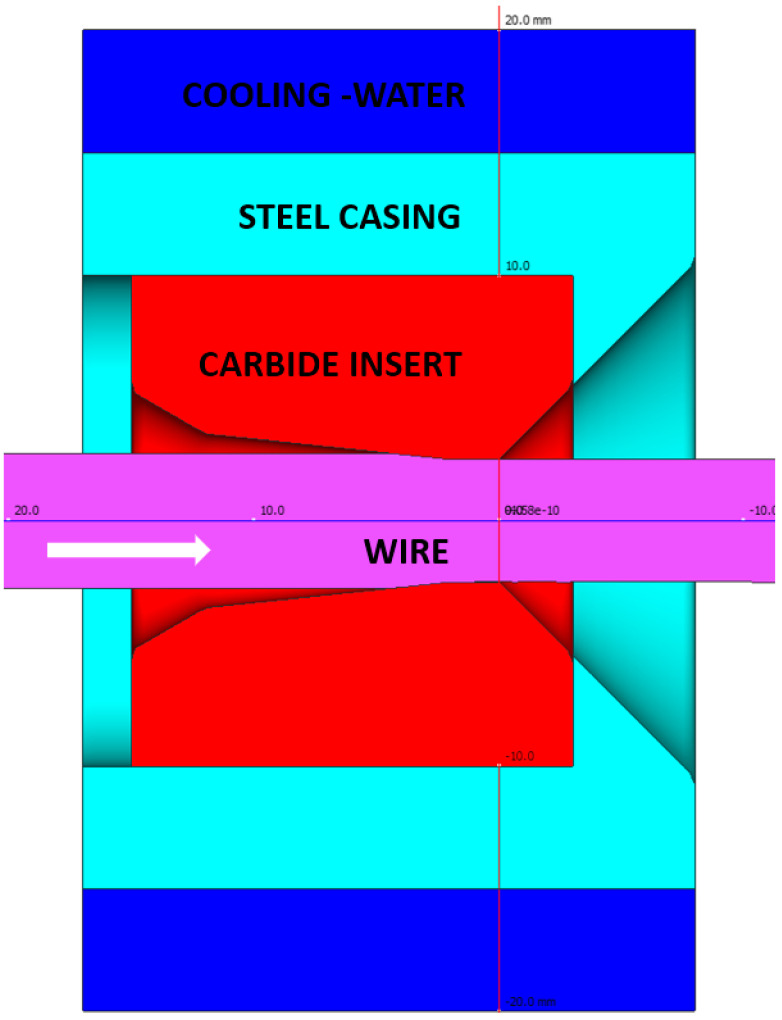
The drawing process tools.

The model adopts an elastic–plastic model of the deformed body (wire), while rigid models with the possibility of calculating heat distribution were adopted for the insert, insert housing, and water (Figure 1).

Determining the wear of the drawing die insert during the drawing process requires the determination of the working conditions of the drawing die as well as the conditions prevailing on the drawing die surface in the area of the crushing cone and the calibrating part.

The following initial parameters and boundary conditions were adopted for the numerical modelling:

- Initial temperature: water 20 °C, drawing die housing 20 °C, insert 20 °C, wire 100 °C;
- Thermal conductivity coefficient: wire insert: 1000 W/(m^2^-K); housing insert: 20,000 W/(m^2^-K); housing water: 10,000 W/(m^2^-K);
- Friction coefficient: 0.07;
- C45 steel properties, Table 1.

For the numerical modelling, it was necessary to determine the remaining physical–mechanical properties of the material:

- Young’s modulus of C45 steel—temperature-dependent;
- Poisson’s number of C45 steel: 0.283;
- Thermal conductivity of C45 steel: temperature-dependent;
- The dependence of K10 carbide hardness on temperature was taken from the paper [26];
- Carbide thermal conductivity: 80 W/(m·K);
- Carbide heat capacity: 0.2 J/(g·K);
- Carbide density: 14,700 kg/m^3^.

**Figure 2:**
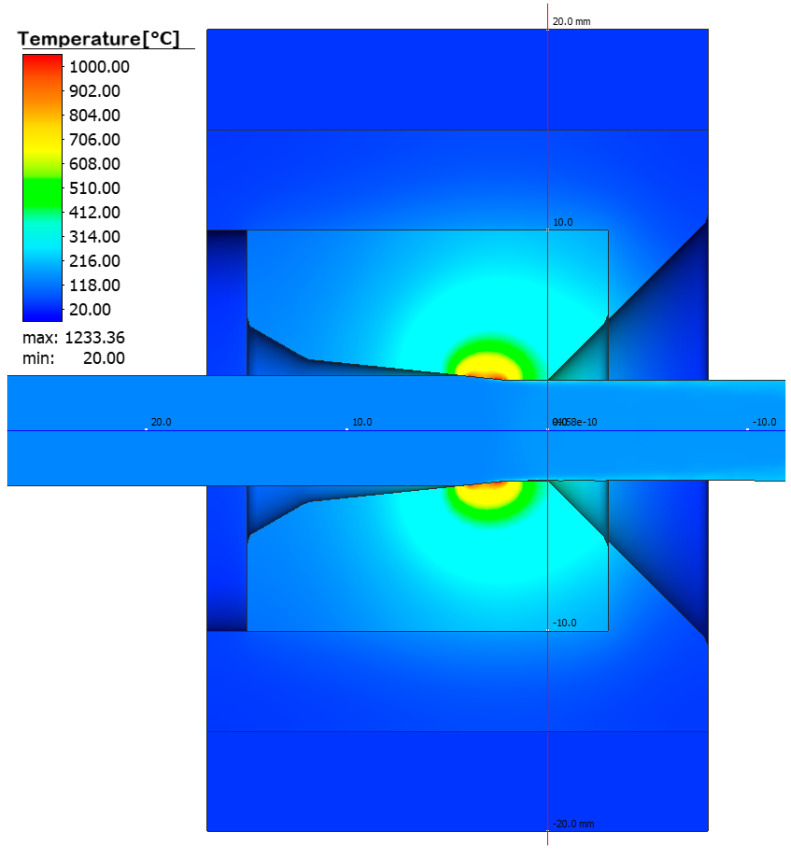
Temperature distribution in the tools during wire drawing at 5 m/s.

**Figure 3:**
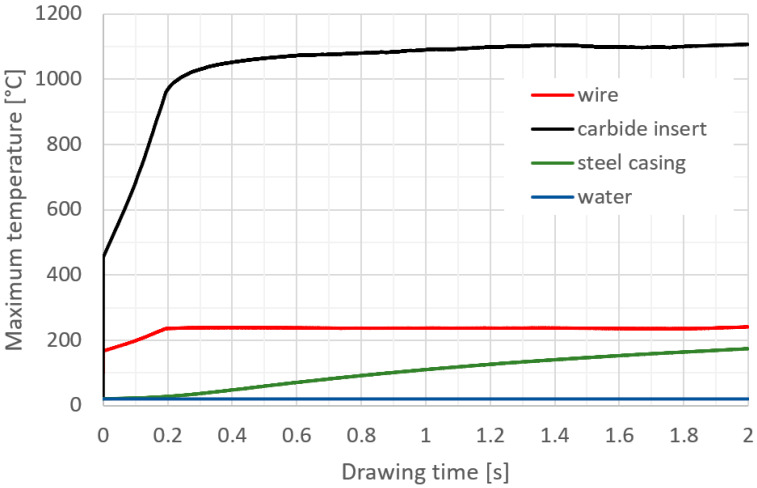
Maximum temperature of drawing die elements after drawing 10 m of wire.

**Figure 4:**
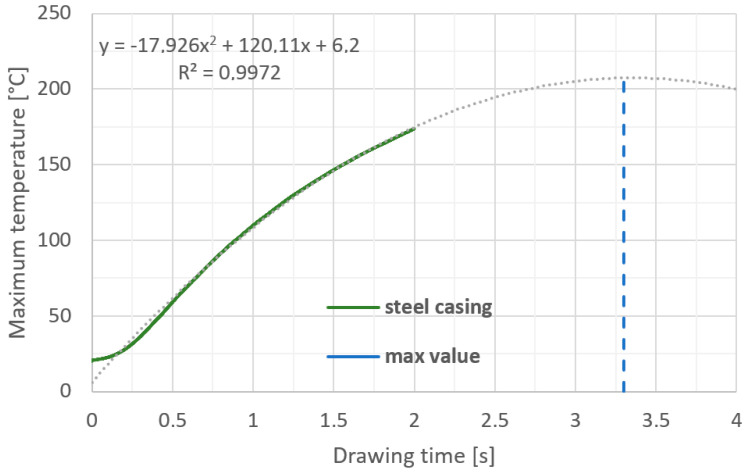
Maximum temperature of steel casing after drawing 10 m of wire.

The purpose of the numerical calculatons was to determine the temperature distribution in a drawing set consisting of a tungsten carbide insert and a C45 steel housing, which is cooled with water on the side of the cylindrical surface. A constant water temperature, 20 °C, was assumed in the calculations due to its circulation in the cooling system. The drawing speed was assumed to be 5 m/s. As a result of the performed modelling, the temperature distribution in the elements of the wire drawing set with an initial diameter of 5.35 mm to a diameter of 5.0 mm was obtained. The drawing process was carried out for a wire length of 10 m and process time of 2 s. Numerical calculations made it possible to determine the temperature distribution in the drawing die and the tool heating rate as a function of drawing time; see Figure 2, Figure 3 and Figure 4.

**Figure 5:**
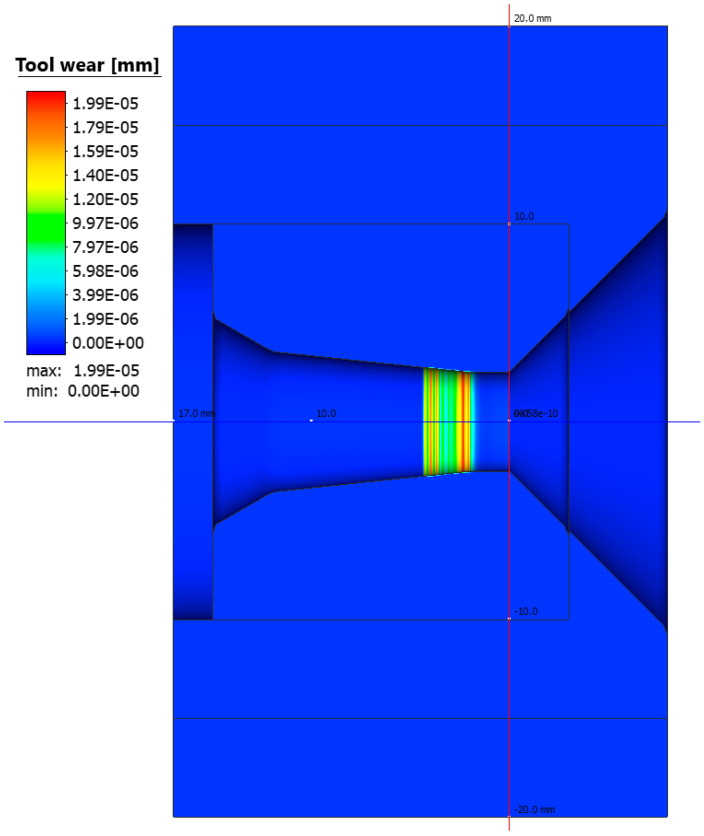
Drawing die wear after drawing 10 m of wire.

Based on the numerical modelling results shown in Figure 3, a clear increase in temperature on the working surface of the drawing die insert is noticeable. In the area 0.06 mm from the working surface of the drawing die, the temperature reaches a value from 950 to 1100 °C. The temperature settles within 1 s and does not increase further in the process. The heat is dissipated through the insert and transferred to the drawing die housing. The drawing die housing is heated by the insert and cooled by water at the same time. Figure 3 and Figure 4 show the temperature rise of the housing. The curve in Figure 4 represents the increase in temperature of the housing up to 2 s of the process. The insert heating curve up to 2 s was extrapolated based on the data. The extrapolation shows that the housing should reach thermal equilibrium at 208 °C within a further 1.4 s. The exact determination of the temperature equilibrium requires further time-consuming calculations. Based on the obtained temperature distribution, it was possible to determine the unit work of friction forces, which makes it possible to determine the quantitative wear of the drawing die. The precise determination of quantitative wear requires determining the wear coefficient. The data shown in Figure 5 are the result of calculations using the Archard model, which describes the abrasive wear of surfaces with a significant difference in speed between the surfaces in contact. That model can be expressed by Equation (5), which is described in the integral form used to solve an FEM-based algorithm.

(5) $$wear=k_{zn}\int_{0}^{t}\frac{\sigma_{n}v_{s}}{H(T)}dt,[\mathrm{mm}]$$

where *v_s_*—tangential sliding velocity of the metal on the tool surface, *σ_n_*—normal stresses, *t*—time, *H*(*T*)—tool hardness at a specified temperature, and *k_zn_*—tool wear coefficient.

Preliminary computer simulations have shown that for a correct representation of the drawing process and the influence of drawing technology (e.g., single and total reduction distributions, tool geometry, drawing speed) on the drawing process and tool wear, it is necessary to assume higher initial tool and material temperatures of 500 °C and 50 °C, respectively. Simulations of wire drawing in a single sequence with the application of different values of a single reduction (Gp = 5.4; 17.4; 25.4; 33.1%) were performed for two drawing speeds of 1 and 5 m/s. The drawing process was carried out using drawing dies with angles 2α = 6°, 10°, and 14°.

### 2.2. Tribological Tests

Tribological tests were carried out as part of this study to determine the influence of temperature on friction conditions and wear of tungsten carbide. Tribological tests were conducted in dry conditions without lubrication. A high-temperature CSM tribometer operating in the Pin–Ball-on-Disk system was used to assess carbide wear (Anton Paar GmbH’s, Graz, Austria). It enables the measurement of tribological properties in the range from ambient temperature to 1000 °C. In the industry, the most commonly used carbide for the manufacture of drawing dies is grade K10 carbide (94%WC+6%Co) [1]. It is used in the drawing of steel wires with carbon content ranging from 0.08%C (low-carbon steel) to 0.9%C (high-carbon steel). Hence, steel samples of grade C45 (the same steel grade as in the computer simulations—Section 2.1) were prepared for tribological testing. The performed carbide abrasive wear tests in a sense reproduce the actual drawing process, during which the wire passing through the drawing die causes its heating and gradual wear. Table 2 presents the parameters of the tribological tests.

Microscopic analyses were carried out using an Olympus GX41 light microscope (Olympus, Tokyo, Japan), a VHX-7000 digital microscope and a scanning electron microscope (SEM). Chemical composition was analysed using scanning electron microscopy SEM+EDS (JEOL Ltd., Tokyo, Japan).

Surface roughness was assessed using a VHX microscope with a Gaussian filter. Based on the performed tests, the following parameters were determined: Sa (arithmetic mean height), Sz (maximum height), Sp (height of highest elevation), Sv (depth of lowest indentation), and Sq (mean squared height).

## 3. Results

### 3.1. Numerical Analysis of the Drawing Process

**Figure 6:**
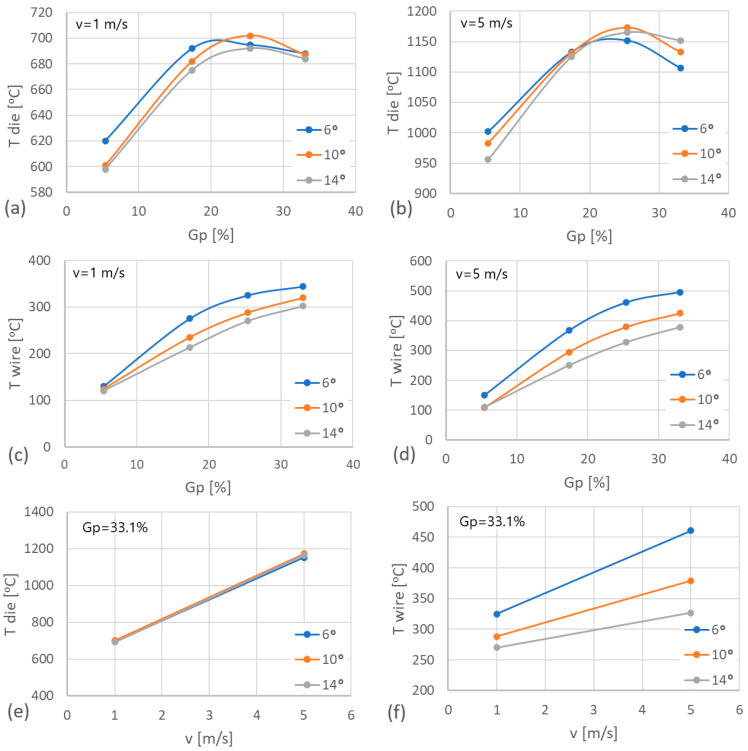
Influence of drawing parameters on the heating of drawing dies and wire: (**a**) die temperature in single reduction function for v = 1 m/s; (**b**) die temperature in single reduction function for v = 5 m/s; (**c**) wire temperature in single reduction function for v = 1 m/s; (**d**) wire temperature in single reduction function for v = 5 m/s; (**e**) die temperature in drawing speed function; (**f**) wire temperature in drawing speed function; where: v—drawing speed; Gp—single reduction; T—temperature; 2α = 6°, 10°, 14°.

Temperature is the main parameter influencing the wear rate of drawing dies in the drawing process. The performed computer simulations made it possible to determine the correlation between the single reduction size, the drawing die angle, the drawing speed, and the temperature of the drawing dies and wire in contact zone; see Figure 6.

The wire and drawing die temperatures depend on the amount of generated heat. In the drawing process, the source of heat is the work of deformation and the work of friction forces. Based on Figure 6, it can be noticed that the temperature of the drawing die increases with an increase in single reduction, it reaches a maximum value, and then it decreases slightly. However, in the case of wire, there is a continuous increase in temperature over the entire range of the discussed single reduction values. Another fundamental parameter in the drawing process influencing the heating of wire and tools is the geometry of the drawing dies. Computer simulations showed that increasing the drawing die angle from 6 to 14° resulted in an increase in the temperature of the drawing dies, while the wire temperature decreased. The described phenomenon can be explained as follows. The drawing angle influences the duration of contact of the wire and the drawing die; the higher the angle value, the lower the friction forces resulting in the heating of the drawing dies. At the same time, the contact duration of the wire and the drawing die decreases. In such conditions, a larger amount of the generated heat is absorbed by the wire and a smaller amount is absorbed by the drawing die. An additional factor causing heat accumulation in the wire-drawing die tribological system is the drawing speed. Increasing the drawing speed from 1 to 5 m/s resulted in a five-fold increase in deformation work per unit of time. Section 2.1 shows that the drawing die is water-cooled during its operation, which increases the heat transfer from the tool. The drawing die housing is heated by the insert and cooled by water at the same time. At a given drawing speed, which depends on the reduction size, tool geometry and wire grade, the temperature of the drawing dies rises sharply, as the heat flow generated by plastic deformation and friction significantly exceeds the heat flow absorbed by the water cooling the drawing die housing. An approximately five-fold increase in drawing speed results in an approximately two-fold increase in the temperature of the drawing dies and wire. An increase in wire temperature has a negative impact on the lubrication conditions, leading to even greater heating of the drawing dies and, in extreme cases, to damage to the drawing die surface and premature wear.

**Figure 7:**
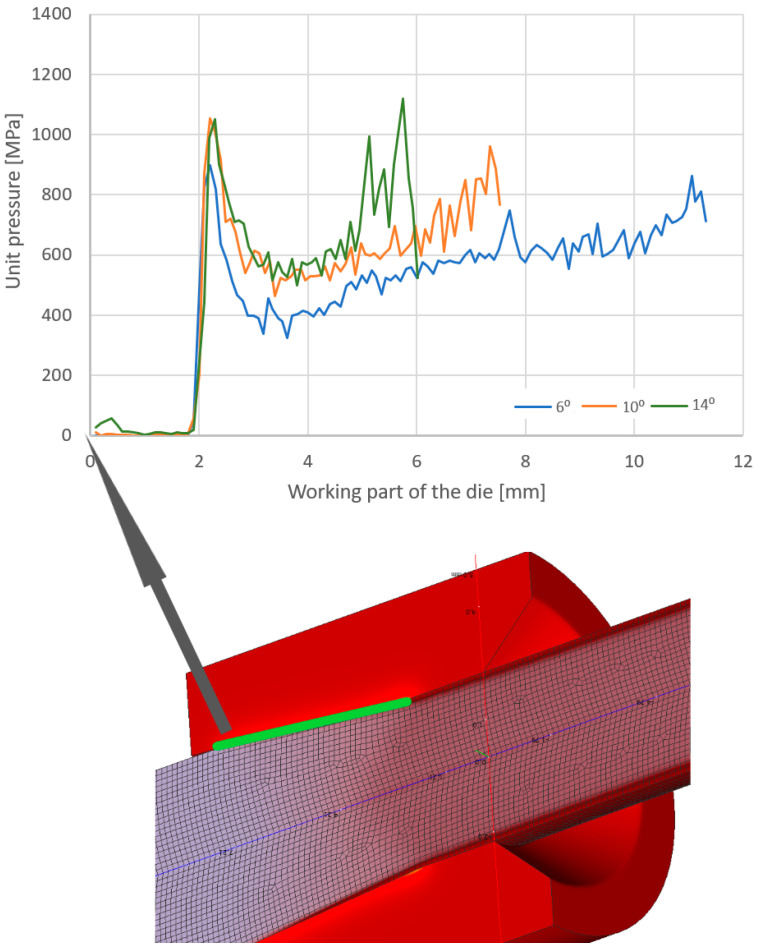
Distribution of unit pressure along the wire/tool contact area in the working part of the drawing die, where 2α = 6°, 10°, 14°.

A parameter that significantly influences tool wear in metal-forming processes is unit pressure. Figure 7 shows the distribution of unit pressure along the wire/tool contact area in the crushing part of the drawing die for angles 2α = 6, 10, 14° (drawing from 5.5 to 4.5 mm).

**Figure 8:**
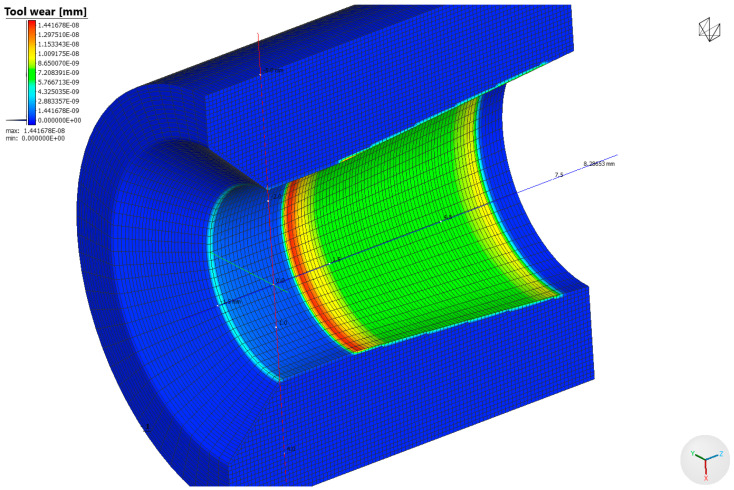
Example of drawing die wear in wire drawing process for drawing die angle 2α = 10°, single reduction Gp = 33%, drawing speed v = 1 m/s.

**Figure 9:**
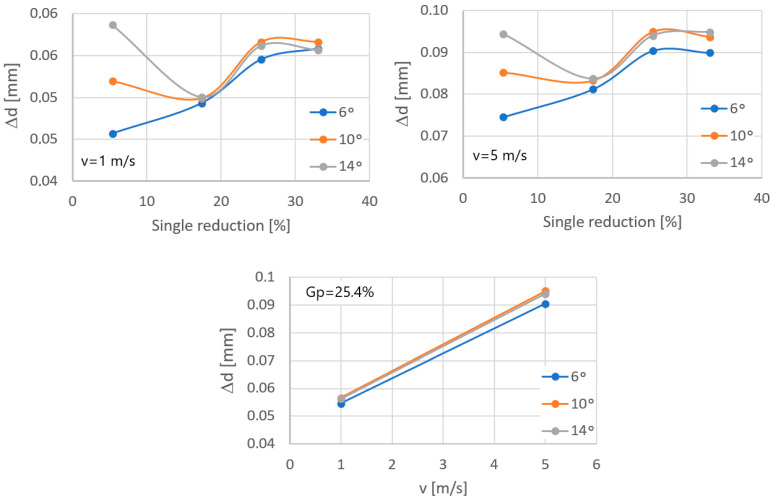
Change in the diameter of the calibrating part of the drawing die after the drawing process, where ∆d—drawing die wear expressed in mm; 2α = 6°, 10°, 14°.

Based on the data in Figure 7, it can be seen that the unit pressure is not a linear function with the highest values at the wire entry into the drawing die and near the wire transition from the crushing part of the drawing die to the calibrating part. Increasing the angle of the drawing die from 6° to 14° increased the unit pressure, depending on the location, by approximately 50%, on average. The unit pressure influences the rate of tool wear. Thus, it can be assumed that a large increase in the pressure in the drawing die will result in its accelerated wear and, in extreme cases, even its damage. Comparing Figure 6 and Figure 7, a certain correlation can be observed. An increase in the drawing angle results in a decrease in the temperature of the wire with a simultaneous increase in the unit pressure. A large drawing angle means lower friction (reduced contact between the rubbing surfaces), decrease in wire temperature and a simultaneous increase in deformation resistance and unit pressure. Depending on which parameter is decisive, the applied particular drawing die and single reduction geometry may result in an increase or decrease in drawing die wear. Therefore, the wear rate of drawing dies after drawing 10,000 kg of wire was determined in the study, and the calculation results are presented in Figure 8 and Figure 9.

Based on the performed analysis of drawing die wear, the optimum drawing angle depends on the single reduction size. The application of large drawing die angles with unit deformations below 10% results in a significant increase in drawing die wear (the difference between the angle of 6 and 14° was over 30%). However, with larger single deformations, the differences in drawing die wear between the analysed variants decrease. According to the authors, this is related to the occurrence of opposing phenomena during drawing, i.e., friction, which depends on the duration of contact of the material and tool, the wire and drawing die temperatures and the pressure on the drawing die. Thus, the degree of drawing tool wear in the wire drawing process is the result of the above-mentioned factors. Industrial practice shows that when designing single reduction distributions to optimise drawing die wear, the applied principle is that the optimal angle of the working part of the drawing die depends on the degree of wire reinforcement and the single reduction size. Hence, in multi-stage drawing, as the wire goes through successive drawing dies, the single reduction value and the angle of the working part of the drawing die decrease.

### 3.2. Results of Tribological Tests

The performed tribological tests confirmed the significant influence of temperature on the friction conditions and wear of the carbide/steel friction pair. Based on the data in Table 3, it can be seen that while an initial increase in temperature to 200 °C does not result in a significant difference in friction coefficient values (a difference of approximately 3%), a considerable deterioration in lubrication conditions can be expected at temperatures above 400 °C. The differences in the values of the average friction coefficient between variants I and IV were over 30%.

**Figure 10:**
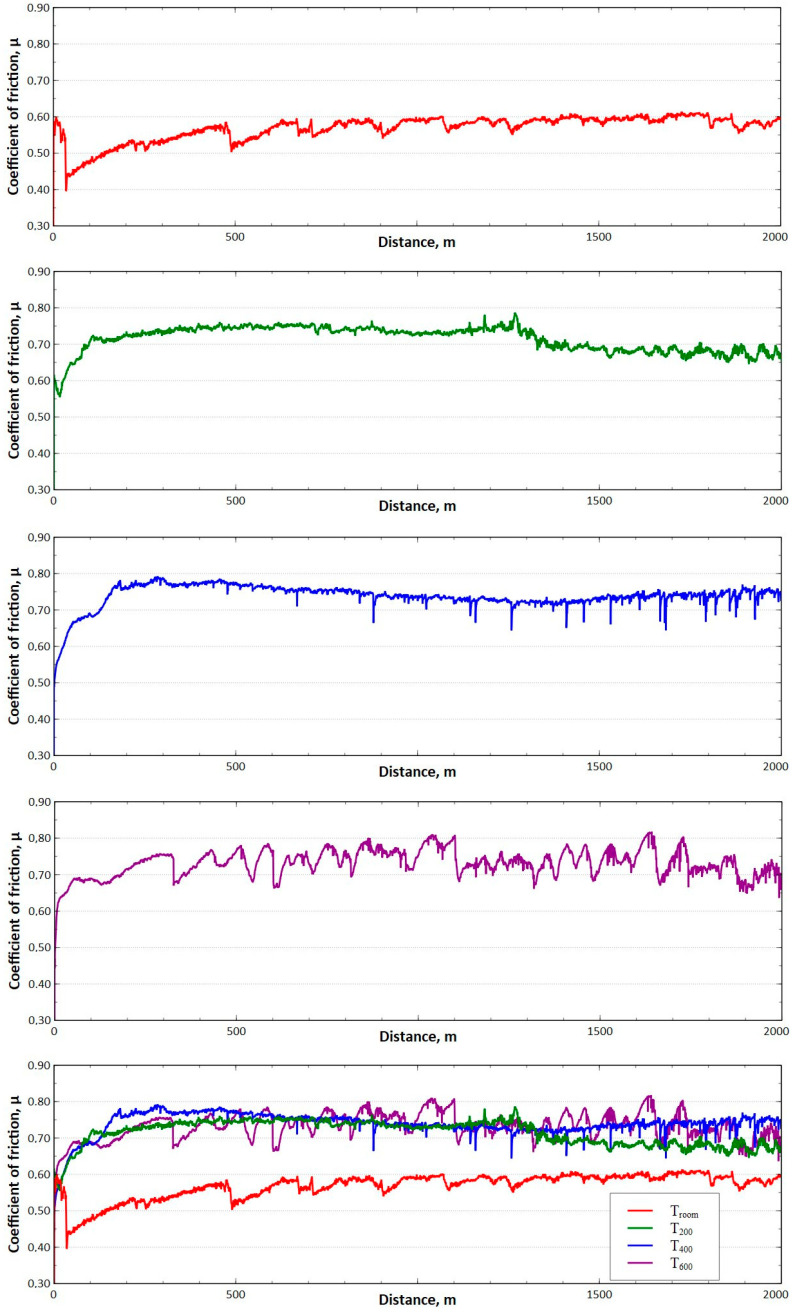
Dependence of the friction coefficient on the friction path (distance) for variants I–IV.

The analysis of the change in the masses of the samples and counter-samples shown in Table 3 is a complex issue. In the performed tests, there was a gradual abrasion of the steel and carbide sample surfaces with the “products” of abrasion sticking to their surfaces. Unlike in the case of tribological tests performed at ambient temperature, when testing at higher temperatures, an additional factor influencing the condition of the surface layer is the phenomenon of scale formation on the surface of the test samples. Hence, among the analysed variants, the highest total change in the sample and counter-sample mass was recorded for variant IV (T = 600 °C). During the testing, pieces of scale are deposited on the rubbing surfaces. Consequently, depending on the resultant of the above-mentioned factors, the value of the friction coefficient is not constant and changes periodically, as shown in Figure 10. The obtained results confirm the hypothesis contained in Section 2 regarding the influence of temperature on the accelerated wear of drawing dies. An increase in the friction value in the process causes an increase in the deformation resistance at the wire/tool interface and accelerates the wear of the drawing dies. Therefore, in the actual drawing process, lubricants are used to reduce friction and to protect the surface of the drawing die from abrasion when the wire is pulled through it. An increase in drawing die temperature during drawing causes a deterioration in the lubricant’s rheological properties, e.g., the dynamic viscosity coefficient, as well as a reduction in the lubricant film thickness in the contact zone. This leads to an even faster wear of the drawing die. Obviously, the tribological tests presented in this paper do not ideally reflect the frictional conditions of the drawing process, especially in industrial drawing (a shorter friction path and lower pressures in the contact zone were implemented in the laboratory tests). Nevertheless, they confirm the role of temperature in the formation of the carbide surface layer and its resistance to abrasion. Hence, as part of the study, metallographic tests of the sample and counter-samples were performed after tribological testing.

The analysis of the surface layer, after tribological testing, consisted of assessing changes in the structure and properties of C45 steel (sample) and WC carbide (counter-sample). In the first stage, the influence of temperature on the structural changes of the sample, outside the abrasion area, was determined.

**Figure 11:**
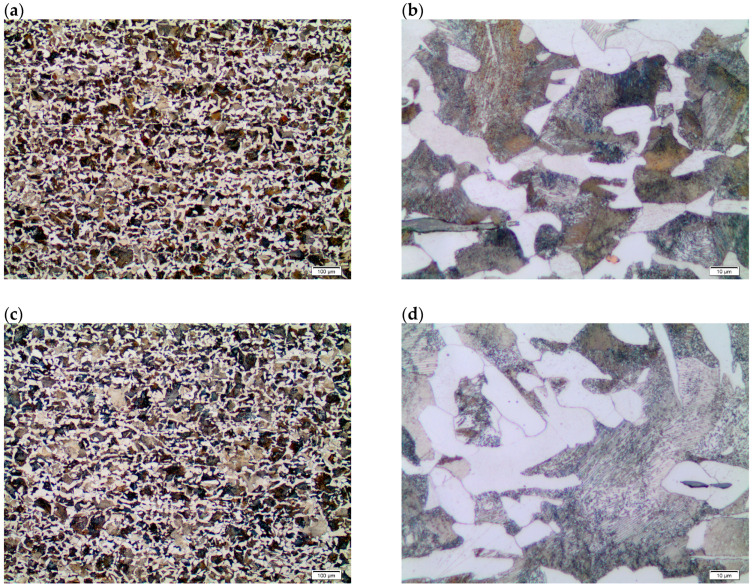
Structure of C45 steel, LM: (**a**) variant I, 100× magnification; (**b**) variant I, 1000× magnification; (**c**) variant IV, 100× magnification; (**d**) variant IV, 1000× magnification.

The obtained microscopic test results, shown in Figure 11, confirm that regardless of the process temperature, the structure of the C45 steel does not change.

For variants I, II, III and IV, a ferritic–perlitic structure with different grain sizes was observed. On the majority of the surface, the perlite was surrounded by a lattice of ferrite (Figure 11a,d). At 1000× magnification, the perlite clearly revealed its lamellar structure, which contained both thicker and thinner lamellae (Figure 11b,d).

**Figure 12:**
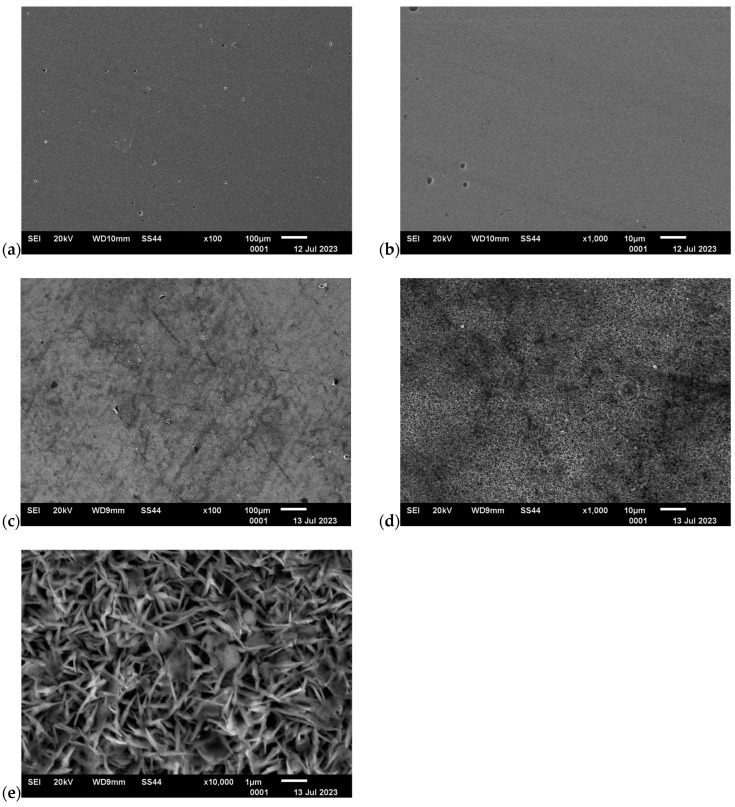
Surface next to the abrasion area, C45 steel, SEM: (**a**) variant I, 100× magnification; (**b**) variant I, 1000× magnification; (**c**) variant IV, 100× magnification; (**d**) variant IV, 1000×; (**e**) variant IV, 10,000× magnification.

The analysis of the surface of the test samples showed that the temperature in the tribological tests contributed to the formation of oxides/deposits on the C45 steel. The appearance of the surface of C45 steel next to the abrasion area is shown in Figure 12. Based on the analysis of the EDS chemical composition, in the case of variant I, only the elements included in C45 steel were determined, i.e., carbon, manganese and iron. In the other cases, in addition to the above-mentioned components, oxygen was also present, which confirms the formation of iron oxides on the steel surface (variants II, III, IV). As part of the microscopic analysis, a significant difference in surface morphology was observed for variant IV compared to variants I, II and III. In this case, the structure has a coniferous form; see Figure 12e.

**Figure 13:**
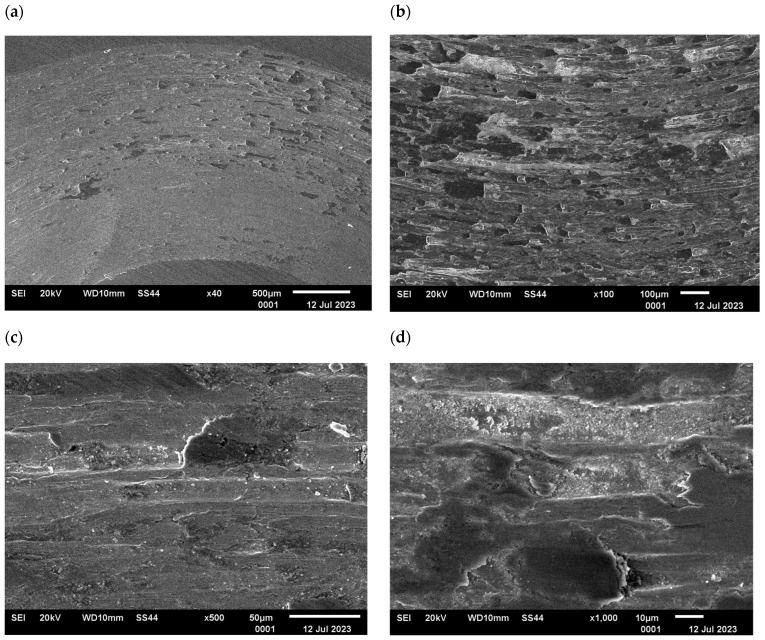
Variant I, C45 steel surface, SEM: (**a**) abrasion area, 40× magnification; (**b**) abrasion area, 100× magnification; (**c**) abrasion area, 500× magnification, (**d**) abrasion area, 1000× magnification.

**Figure 14:**
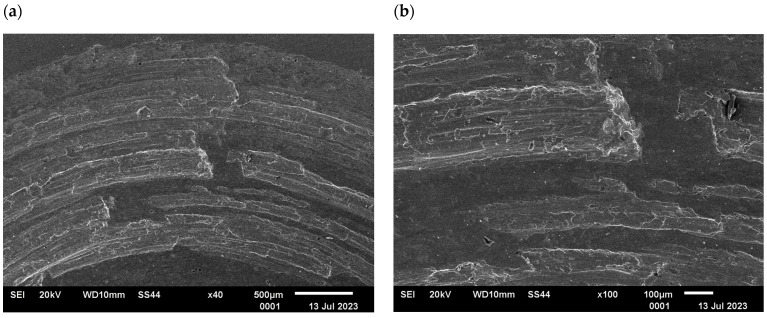

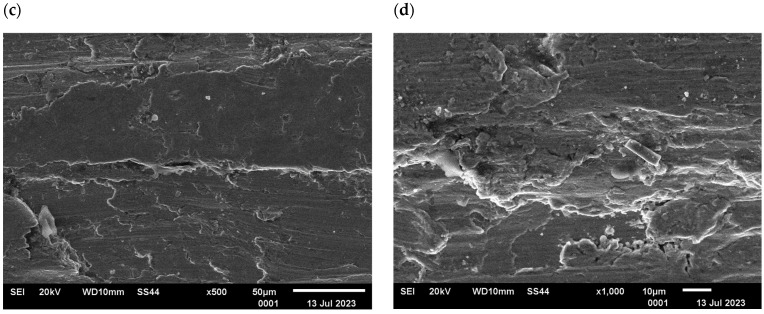
Variant II, C45 steel surface, SEM: (**a**) abrasion area, 40× magnification; (**b**) abrasion area, 100× magnification; (**c**) abrasion area, 500× magnification, (**d**) abrasion area, 1000× magnification.

**Figure 15:**
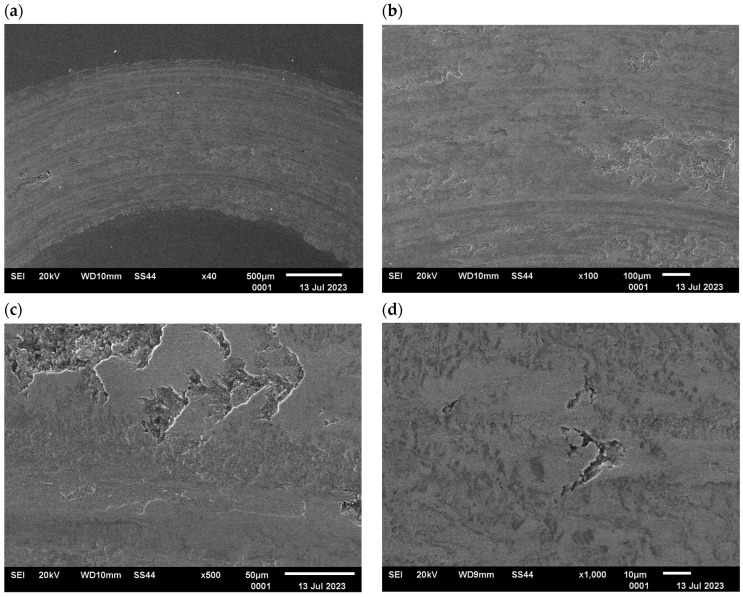
Variant III, C45 steel surface, SEM: (**a**) abrasion area, 40× magnification; (**b**) abrasion area, 100× magnification; (**c**) abrasion area, 500× magnification, (**d**) abrasion area, 1000× magnification.

**Figure 16:**
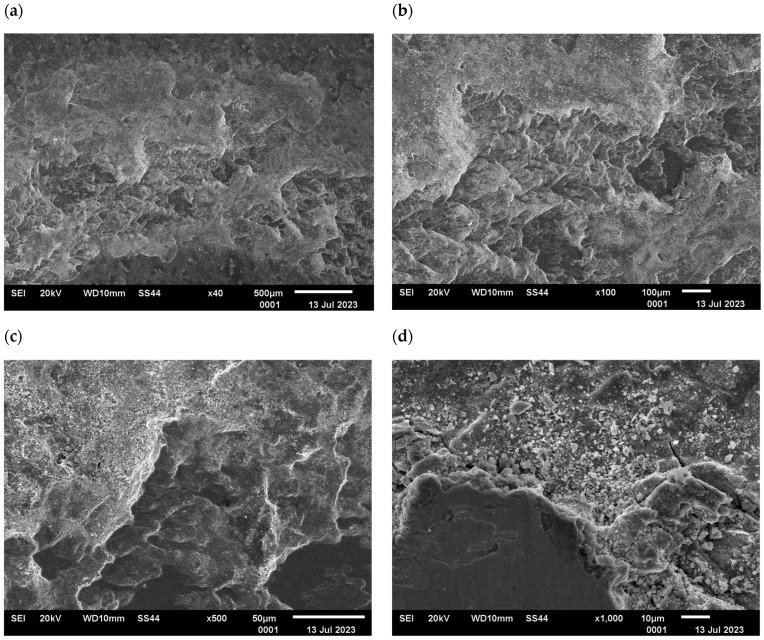
Variant IV, C45 steel surface, SEM: (**a**) abrasion area, 40× magnification; (**b**) abrasion area, 100× magnification; (**c**) abrasion area, 500× magnification, (**d**) abrasion area, 1000× magnification.

**Figure 17:**
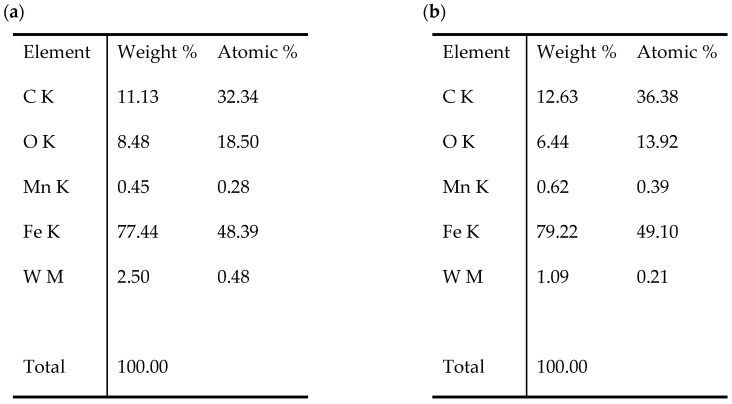

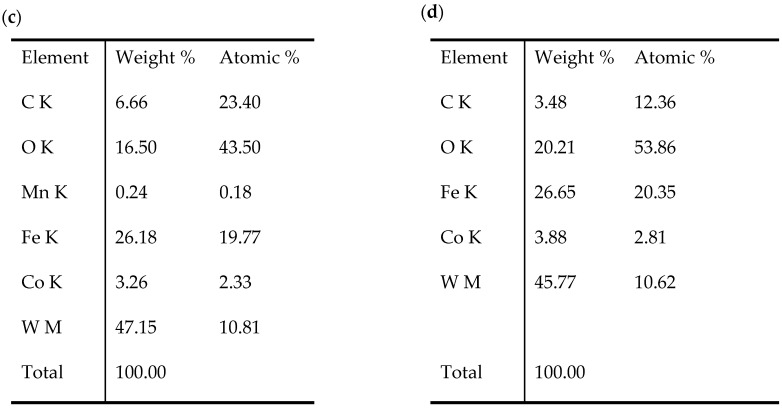
EDS analysis, C45 steel, abrasion area: (**a**) variant I, (**b**) variant II, (**c**) variant III, (**d**) variant IV.

The changes in the surface layer of the steel samples indicate that the temperature during tribological testing has a significant influence on the abrasion area. The results of the microscopic analysis and the chemical composition at the abrasion area for the different variants are presented in Figure 13, Figure 14, Figure 15, Figure 16, and Figure 17, respectively.

**Figure 18:**
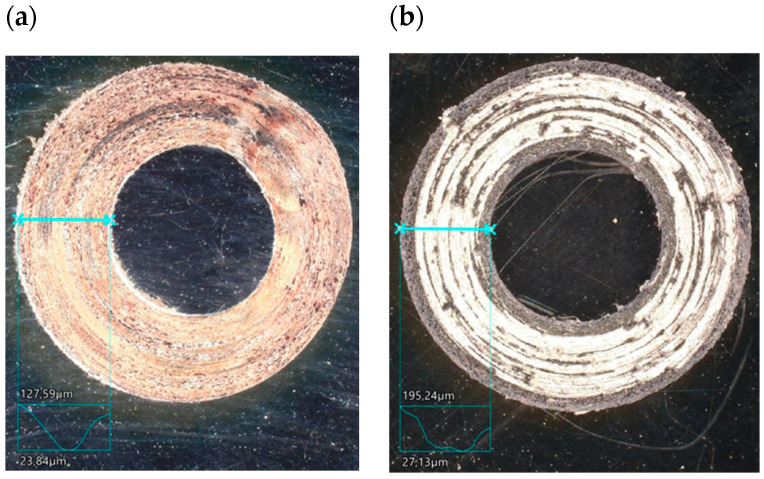

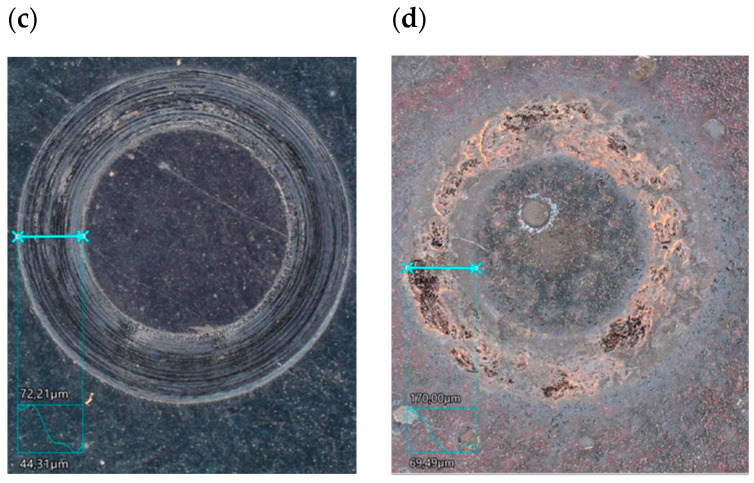
Image of C45 steel abrasion area, digital microscope: (**a**) variant I, (**b**) variant II, (**c**) variant III, (**d**) variant IV.

**Figure 19:**
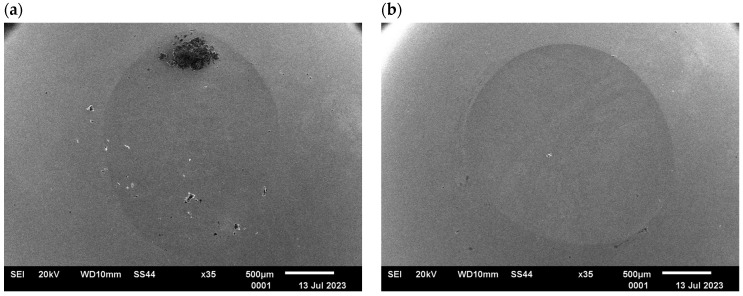

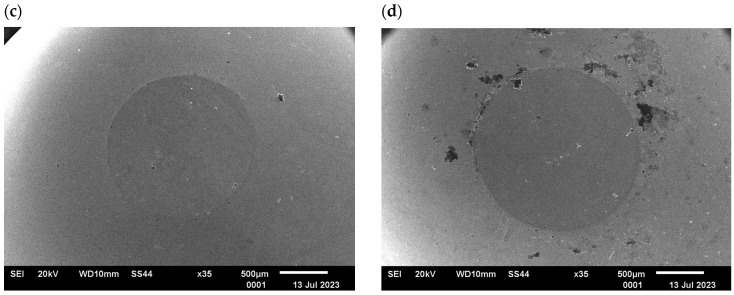
Image of ball abrasion area, SEM: (**a**) variant I, (**b**) variant II, (**c**) variant III, (**d**) variant IV.

The study showed significant differences in the abrasion area between the variants. In the case of variant I, there is more damage on the outer side of the abrasion area. Significant material loss is noticeable in that place. Variant II, in turn, is characterised by more uniform abrasion along the entire length of the abrasion radius. In the case of sample III, pitting occurs locally in the abrasion area, while variant IV is characterised by the formation of deposits on the surface, which is probably due to the higher process temperature. Analyses of the chemical composition in the abrasion area showed (Figure 17) that in the case of processes I and II, oxygen and tungsten were present in addition to the elements that made up C45 steel, such as C, Mn and Fe. In those variants, the amount of observed tungsten did not exceed 2.5% by weight. However, for variants III and IV, its proportion increased dramatically and exceeded 45% by weight. Moreover, the presence of cobalt—in the amount of 3% by weight—was recorded in those variants. The presence of tungsten and cobalt in the abrasion area was due to the fact that material from the counter-sample (ball) was deposited onto the steel (“rubbed into”). Further analysis showed significant differences in abrasion width and depth for the individual variants (Figure 18 and Figure 19, Table 4). Microscopic observations enabled noticing irregularities in abrasion marks. This was most visible for variant I. The largest abrasion mark was also observed in this case (the measurement was taken at the widest point).

For variant II, the abrasion area width was approximately 22 units smaller than for variant I, while the depth was over 64 units greater in this case. For variant III, the width and depth of the abrasion area dropped to approximately 1500 μm and 28 μm, respectively. For variant IV, in turn, the width and depth of the abrasion area increased (~1708 units—first parameter, ~100 units—second parameter).

**Figure 20:**
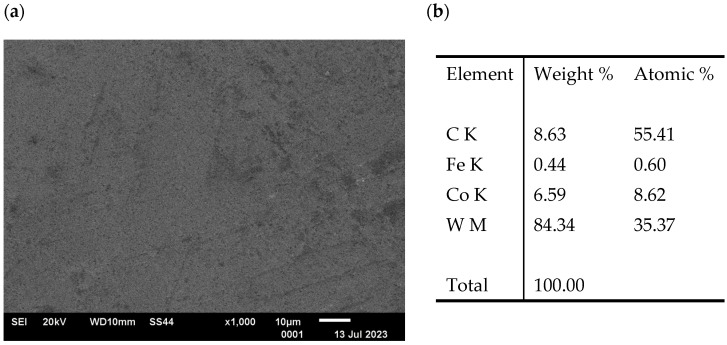
Image of the ball abrasion area (**a**) and chemical composition (**b**) for the variant III sample.

The analysis of the surface of the counter-samples (balls) confirmed the presence of typical abrasion marks; however, no surface cracks were found. The performed tests showed that in the contact area of the sample/counter-sample during tribological testing, the phenomenon of sample material deposition on the surface of the counter-sample occurs; see Figure 20. This confirms the possibility of the steel “sticking” to the surface of the drawing dies during the drawing process.

**Figure 21:**
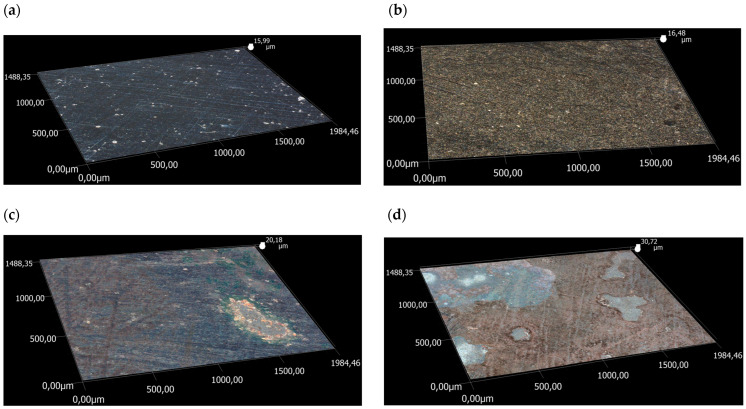
Three-dimensional (3D) image steel simples after tribological tests for (**a**) variant I, (**b**) variant II, (**c**) variant III, and (**d**) variant IV.

The parameter that influences the rate of tool wear is the surface topography. The following parameters were selected to be analysed: arithmetic mean surface roughness Sa, root mean square roughness Sq, ten-point height of irregularities surface Sz (mean absolute height of the five highest and five lowest vertex cavities), the maximum height of the elevation surface Sp (the distance from the highest point of the mean plane), and the maximum depth of the cavity surface Sv (the distance from the lowest point of the mean plane). Hence, the influence of temperature on the surface topography of steel samples was determined in this study. The measurement results are shown in Figure 21 and Table 5.

The performed tests proved the influence of the test temperature on the surface topography of the steel samples, indicating that the roughness profile deviations increase. There are discrepancies in the values of the Sa, Sq, Sz, and Sp, and based on the data shown in Figure 21, as the temperature increases, the height parameters and Sv parameters between variants I and IV were approximately 100%. The increase in surface roughness, especially for variant IV, is associated with the oxidation processes of the steel surface during tribological testing. Surface roughness has a strong influence on friction conditions. The final stage in the manufacture of drawing tools is polishing, which is performed to obtain a smooth surface on the drawing dies. As more wire is drawn through, the surface of the drawing dies becomes increasingly rough, which leads to increased friction and accelerated wear. This is confirmed by surface topography tests on steel samples, which prove that there is a correlation between surface roughness and friction coefficient (Figure 10 and Figure 21).

## 4. Conclusions

Numerical analysis has shown that after drawing approximately 10 m of wire, the temperature of the tungsten carbide insert stabilises, and its value depends on the drawing process parameters, i.e., single reduction, drawing die angle, and drawing speed.

An increase in drawing angle results in a decrease in wire temperature with a corresponding increase in unit pressure. A large drawing angle means lower friction (reduced contact between the rubbing surfaces), decrease in wire temperature, and a simultaneous increase in deformation resistance and unit pressure. Depending on which parameter is decisive, the applied particular drawing die and single reduction geometry may result in an increase or decrease in drawing die wear.

Based on the performed analysis of drawing die wear, the optimum drawing angle depends on the single reduction size. The application of large drawing die angles with unit deformations below 10% results in a significant increase in drawing die wear (the difference between the angle of 6 and 14° was over 30%).

The wear rate of drawing dies in wire drawing is the result of friction, which depends on the duration of contact of the material and tool, the wire and drawing die temperatures and the pressure on the drawing die.

It has been shown that during tribological testing, there was a gradual abrasion of the steel and carbide sample surfaces with the “products” of abrasion sticking to their surfaces. Analysis of the chemical composition at the steel abrasion area showed that the share of tungsten and cobalt increases with increasing temperature. At 400 °C, it is more than 45% for tungsten and 3% for cobalt. At higher temperatures, of approximately 600 °C, an increase of about 30% in the friction coefficient was observed.

The analysis of the surface of the counter-samples (tungsten carbide) confirmed that in the sample/counter-sample contact area during tribological testing, the phenomenon of sticking of the sample material (steel) to the surface of the counter-sample (tungsten carbide) occurs, which confirms the possibility of sticking of the steel to the surface of the drawing die during the drawing process.

The research conducted in this work has shown a significant effect of temperature on the friction conditions and abrasive wear of WC+Co sintered carbide. Hence, in the next works, the authors will focus on searching for new materials for drawing cores. The abrasion resistance of dies made of different materials will be compared.

## Figures and Tables

**Table 1:** Yield stress function coefficients (4) for C45 steel/MPa.

Coefficient Values							
*C*_1_	*C*_2_	*m*_1_	*m*_2_	*l*_1_	*l*_2_	*n*_1_	*n*_2_
692.2	−0.00064	0.000028	0.000709	−0.0000473	−0.01224	−0.00031	0.162026

**Table 2:** Tribological test parameters.

Parameter	Variant I	Variant II	Variant III	Variant IV
Counter-sample	WC			
Sampling frequency	60 Hz			
Abrasion radius	3 mm			
Load	20 N			
Linear velocity	50 cm/s			
Friction path	13,000 m			
Time	26,000 s			
Process temperature	ambient	200 °C	400 °C	600 °C

**Table 3:** Mean friction coefficient with mass loss of the rubbing pair.

Material	Mean Friction Coefficient		Mass Loss/Gain, g	
	Mean Friction Coefficient	Standard Deviation	Samples	Counter-Samples
Variant I	0.5642	0.0282	0.0141	0.0027
Variant II	0.5466	0.1101	0.0115	0.0041
Variant III	0.6729	0.0541	+0.0051	0.0013
Variant IV	0.7572	0.0542	+0.0372	0.0020

**Table 4:** Results of C45 steel and ball interaction; W—abrasion width, D—abrasion depth.

Variant I			Variant II			Variant III			Variant IV		
Steel		Ball	Steel		Ball	Steel		Ball	Steel		Ball
W, μm	D, μm	D, μm	W, μm	D, μm	D, μm	W, μm	D, μm	D, μm	W, μm	D, μm	D, μm
2215	103	2249	2193	168	2197	1499	27	1498	1708	100	1716

**Table 5:** Roughness measurement results.

Parameter	Variant I	Variant II	Variant III	Variant IV
Sa, μm	2.49	3.40	3.21	3.91
Sq, μm	13.03	3.99	3.89	4.81
Sz, μm	15.96	16.41	19.89	30.72
Sp, μm	5.82	6.20	8.66	18.85
Sv, μm	10.14	10.21	11.24	11.87

## Data Availability

The original contributions presented in the study are included in the article, further inquiries can be directed to the corresponding author.
